# Inter-visit measurement variability of conjunctival vasculature and circulation in habitual contact lens wearers and non-lens wearers

**DOI:** 10.1186/s40662-019-0135-4

**Published:** 2019-04-01

**Authors:** Jianhua Wang, Liang Hu, Ce Shi, Hong Jiang

**Affiliations:** 1 Miller School of Medicine, Bascom Palmer Eye Institute, University of Miami, 1638 NW 10th Avenue, McKnight Building - Room 202A, Miami, FL 33136 USA; 2 School of Ophthalmology and Optometry, Wenzhou Medical University, Wenzhou, Zhejiang, China

**Keywords:** Variability, Bulbar conjunctiva, Blood flow velocity, Microvascular network, Functional slit-lamp biomicroscopy (FSLB), Hemodynamics

## Abstract

**Background:**

The inter-visit variation of measuring bulbar conjunctival microvasculature and microcirculation needs to be considered when the results from multiple visits are interpreted. This study examined the inter-visit variability of measuring conjunctival microvasculature and microcirculation in habitual contact lens (HCL) wearers and non-contact lens (NCL) wearers.

**Methods:**

Twenty-eight subjects were recruited including 13 HCL wearers (10 females and 3 males; mean age ± standard deviation, 25.8 ± 4.6 years) who had worn contact lenses on a daily basis for at least 3 years and 15 NCL wearers (10 females and 5 males, age 25.5 ± 4.0 years) were recruited. The temporal bulbar conjunctiva was imaged using a functional slit-lamp bio-microscope (FSLB) imaging system. FSLB imaging was performed in the morning when the HCL wearers did not wear their lenses. The measurements included conjunctival vessel diameter, vessel density, blood flow velocity and flow volume. In addition, conjunctival microvasculature was analyzed using monofractal (Dbox, representing vessel density) and multifractal (D0 representing vessel complexity) analyses. The repeated measurement was conducted at least one week after the first visit and both eyes of each participant were imaged. The coefficient of variation (CV) was calculated as the standard deviation of the differences between test and re-test then divided by the mean of the measurements. The intra-class correlation coefficient (ICC) was also calculated.

**Results:**

No significant differences of all vascular measurements in both the right and left eyes were found between two groups (*P* > 0.05). Between two measurements on two different visits, the CV was from 2.4% (vessel density D0) to 63.5% (blood flow volume Q) in HCL wearers and from 3.4% (D0) to 40.6% (blood flow volume) in NCL wearers. The ICC was from 0.60 (vessel diameter) to 0.81 (axial blood flow velocity VA) in HCL wearers and from 0.44 (Q) to 0.68 (cross-sectional blood flow velocity VS) in NCL wearers.

**Conclusions:**

The measurement variability of the vessel density of the bulbar conjunctiva appeared to have the smallest inter-visit variation. The measurement variability of the vasculature and circulation in HCL wearers were similar to that in NCL wearers.

## Background

When a contact lens is placed on the ocular surface, alterations to the vasculature take place immediately in response. Contact lens wear induces ocular surface indentation, especially on the conjunctiva, which causes mechanical pressure to the ocular surface. Wearing a contact lens can disturb the integrity of the tear film and forms two distinct tear films, one between the lens and eye, and another above the lens, which further induces possible friction on the ocular surface. In addition, contact lens wear induces some level of low oxygen supply, although lenses with high oxygen transmission materials (i.e., silicone hydrogel) [1–3] may already eliminate the issue with daily wear of contact lenses. When the eyelid is open, oxygen from the ambient air diffuses into the bulbar conjunctival microvasculature, which highly oxygenates all exposed micro-vessels within 10 s [4]. However, the situation may change if the contact lens covers some portion of the conjunctiva, resulting in vascular responses. In neophytes who have never had experience with contact lenses, contact lens wear induced a 30% increase of bulbar conjunctival blood flow velocity after 6 h of lens wear [5]. Bulbar conjunctival blood flow velocity in habitual lens wearers was about 22% higher than normal controls when they wore their lenses [6]. The alteration of the vessel density (Dbox) in the bulbar conjunctiva in habitual lens wearers was found to be about 3%, which was significant compared to controls. As noted from our previous study, these habitual lens wearers did not notice that their bulbar conjunctivas appeared to be slightly red. These subtle changes may indicate an ongoing response to contact lens wear. Therefore, a precise evaluation of the vascular responses to contact lens wear is critical for studying modern contact lenses. There is a knowledge gap in the inter-visit measurement variation, which is essential for calculating sample sizes and study designs of clinical research on contact lenses.

With the introduction of new contact lens materials (e.g., silicone hydrogel) and lens designs, contact lens-related complications have been dramatically reduced [7–11]. However, some contact lens-associated changes on the ocular surface such as conjunctival hyperemia, corneal infiltration and microbial keratitis often occur [8, 9, 12–15]. While the attention has been shifted to ocular comfort [16], vascular responses to contact lens wear is still a hot topic which can provide better understanding of the underlying mechanism of ocular discomfort associated with contact lens wear [5, 6, 11, 17–20]. Previous evaluation using slit-lamp photography for evaluating the redness of the bulbar conjunctiva was found to have poor measurement variation [2, 3, 21]. Indeed, bulbar conjunctival hyperemia is an obvious sign of vascular response to contact lens insertion [2, 11, 18, 22, 23]. In addition to bulbar conjunctiva redness, blood flow in the vessels alters in concert with the vessel network change, which provides an opportunity for studying the circulation response to contact lens wear [5, 6, 19, 20]. The alterations of conjunctival blood flow velocity in contact lens wearers have been well documented in previous studies [5, 6, 19]. Precise measurements of conjunctival microvasculature (i.e., vessel diameter and density) and microcirculation (i.e., blood flow velocity and flow rate) are crucial in evaluating physiological responses to contact lenses, which often require measurements to be taken during multiple visits on different days [20]. Therefore, the inter-visit variation of these measurements needs to be considered when the results from multiple visits are interpreted. The goal of this study was to examine the inter-visit variability of measuring conjunctival microvasculature and microcirculation in habitual contact lens (HCL) wearers and non-contact lens (NCL) wearers.

## Methods

This study was approved by the Human Subject Research Office (HSRO) at the University of Miami (ID: 20150359) and every participant signed an informed consent form. The study was conducted in accordance with the tenets of the Declaration of Helsinki. Similar to the system used in previous studies by others [17, 24–26], a functional slit-lamp biomicroscope (FSLB) was developed and fully validated [27]. FSLB has been used in previous studies and the procedures are well described [5, 19, 27, 28]. The system applies a high-speed camera which has a video cropping function that enables the addition of a 7× digital magnification into the slit-lamp. This allows a total magnification of up to 210×, which is sufficient for imaging a cluster of red blood cells. With high-speed video capturing, blood flow can be recorded and analyzed to obtain blood flow velocity and flow volume. Since blood flow velocity is different in arterioles [25, 29] and venules [6, 26, 30], conjunctival venules were measured in the present study. Image processing procedures described in the previous studies were used to process the video clips [5, 19, 26–28, 30]. The measurements included axial blood flow velocity (VA), cross-sectional blood flow velocity (VS) and flow volume (Q). The VA was measured by tracking the movement of red blood cells along the vessel [26]. The VS was calculated from VA, using a previously defined function that accounts for the diameter of the blood vessel (D) [30]. The cross-sectional flow rate, Q, was determined using a standard flow rate equation: Q = VS π D^2^/4 [30].

**Fig. 1:**
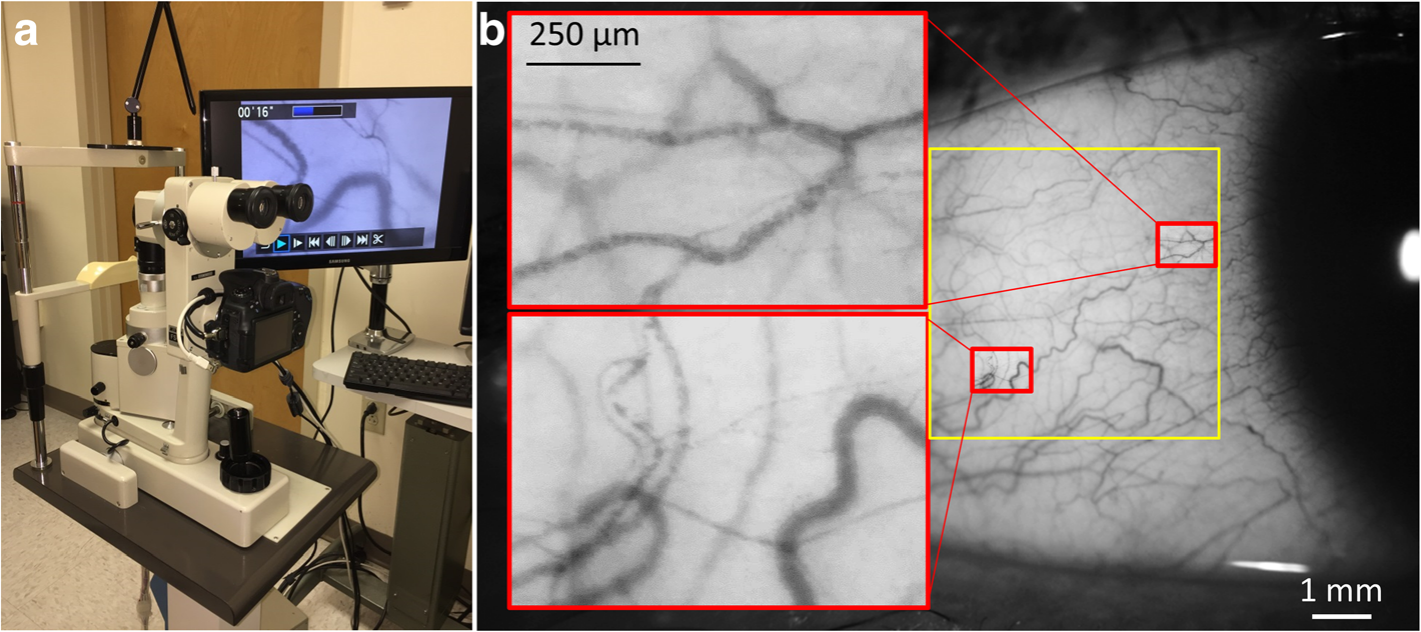
Functional slit-lamp biomicroscope (FSLB) and images. FSLB was modified from a standard slit-lamp biomicroscope equipped with a digital camera through an inherent camera port (**a**). The video cropping function of the camera enables an additional 7× magnification, which is combined with the 30× inherent magnification. FSLB was used to acquire an image of the temporal conjunctiva (**b**) with high magnification, and the red blood cell clusters are shown in the inserts

**Fig. 2:**
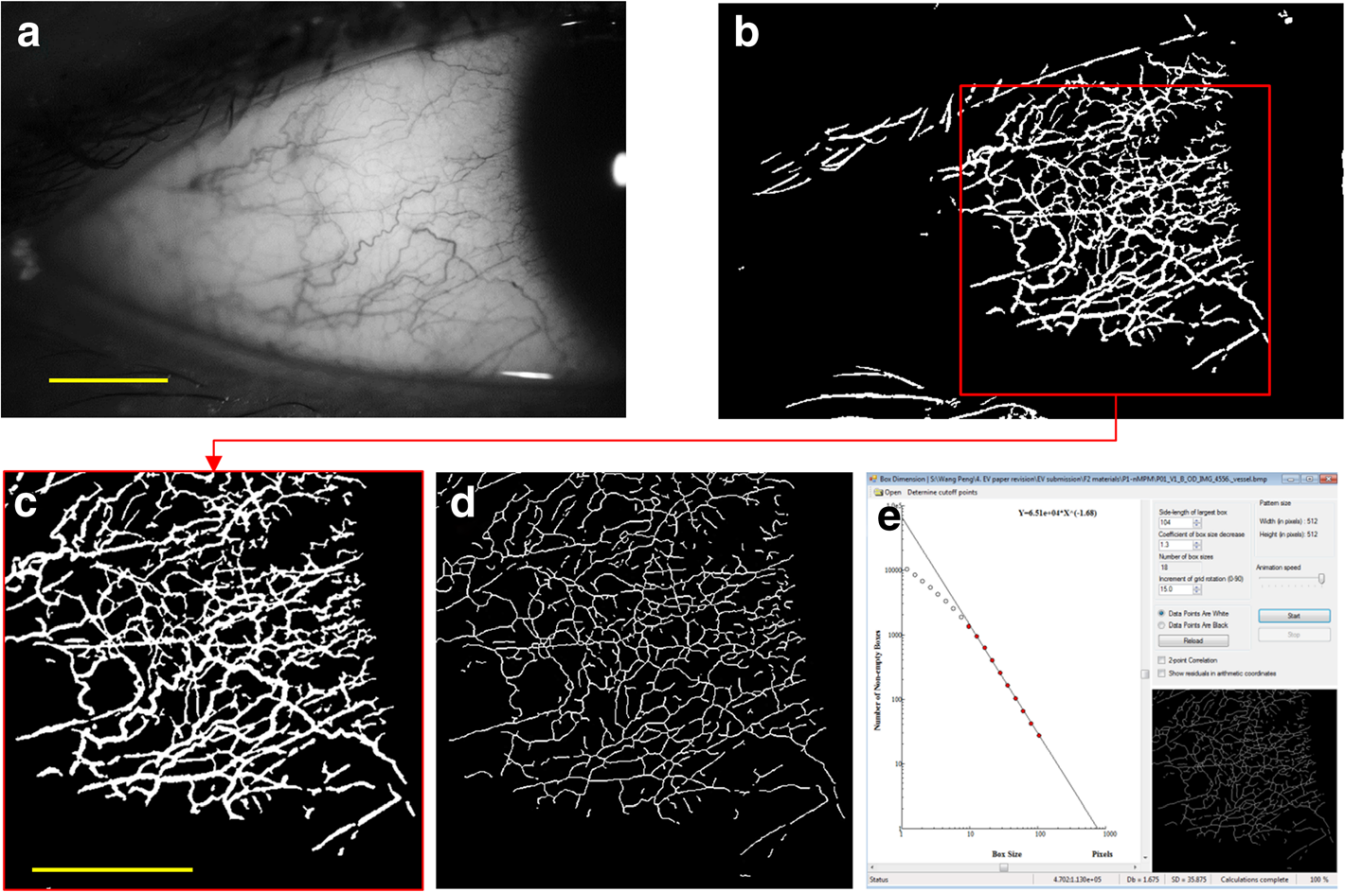
Image processing of conjunctival vasculature and fractal analysis. Conjunctival vessels were segmented by using custom software to extract the conjunctival vessels for fractal analysis. The raw image was resized to 1024 × 683 pixels (**a**). Segmented vessels after using adaptive histogram equalization and morphological opening operations (**b**). Cropped image of 512 × 512 pixels with a field of view 7.87 × 7.87 mm^2^ (**c**). Skeletonized vessel image (**d**). Fractal analysis of the skeletonized image (**e**). Bars = 3 mm

To measure vascular network density, a field of view of 0.9 × 0.7 mm^2^ was used, and six fields of the temporal bulbar conjunctiva located ~ 1 mm radially from the circumference of the limbus ring were imaged for measuring circulation (Fig. 1). In addition, vascular network in a field of 7.87 × 7.87 mm^2^ was acquired (Fig. 2). Image processing procedures extracted the vessels which were skeletonized for fractal analysis using custom software which has been described previously [27]. The monofractal and multifractal analyses available in a software program (Benoit™, TruSoft Inc., St. Petersburg, FL, USA) [27] were performed to evaluate the vessel density (Dbox,) and complexity (D0) of the conjunctival microvasculature. Fractal dimension has been used to estimate the branching pattern of the retinal vascular tree [31] and capillary network in the retina and conjunctiva [27, 32]. Larger values indicate more dense vessels and more complex branching patterns.

Thirteen HCL wearers (10 females and 3 males; mean age ± standard deviation, 25.8 ± 4.6 years) who had worn contact lenses on a daily basis for at least 3 years and 15 NCL wearers (10 females and 5 males, age 25.5 ± 4.0 years) were recruited. FSLB imaging was performed in the morning (from 8:30 am to 11 am) when the HCL wearers did not wear their lenses. The repeated measurement was conducted at least one week after the first visit and both eyes of each participant were imaged.

All data management and statistical analyses were carried out in Excel (v. 2010, Microsoft, Redmond, WA). The coefficient of variation (CV) was calculated as the standard deviation of the differences between test and re-test then divided by the mean of the measurements. The Bland-Altman plot was used show the limits of agreement. The intra-class correlation coefficient (ICC) was also calculated using the Pearson correlation. The confidence interval of the CV was calculated as the CV ± 1.96 × CV/square root (2 × sample size). The student’s t-test was used to test the differences of vascular measurements between groups. A *p*-value of less than 0.05 (*P* < 0.05) was considered statistically significant.

## Results

**Fig. 3:**
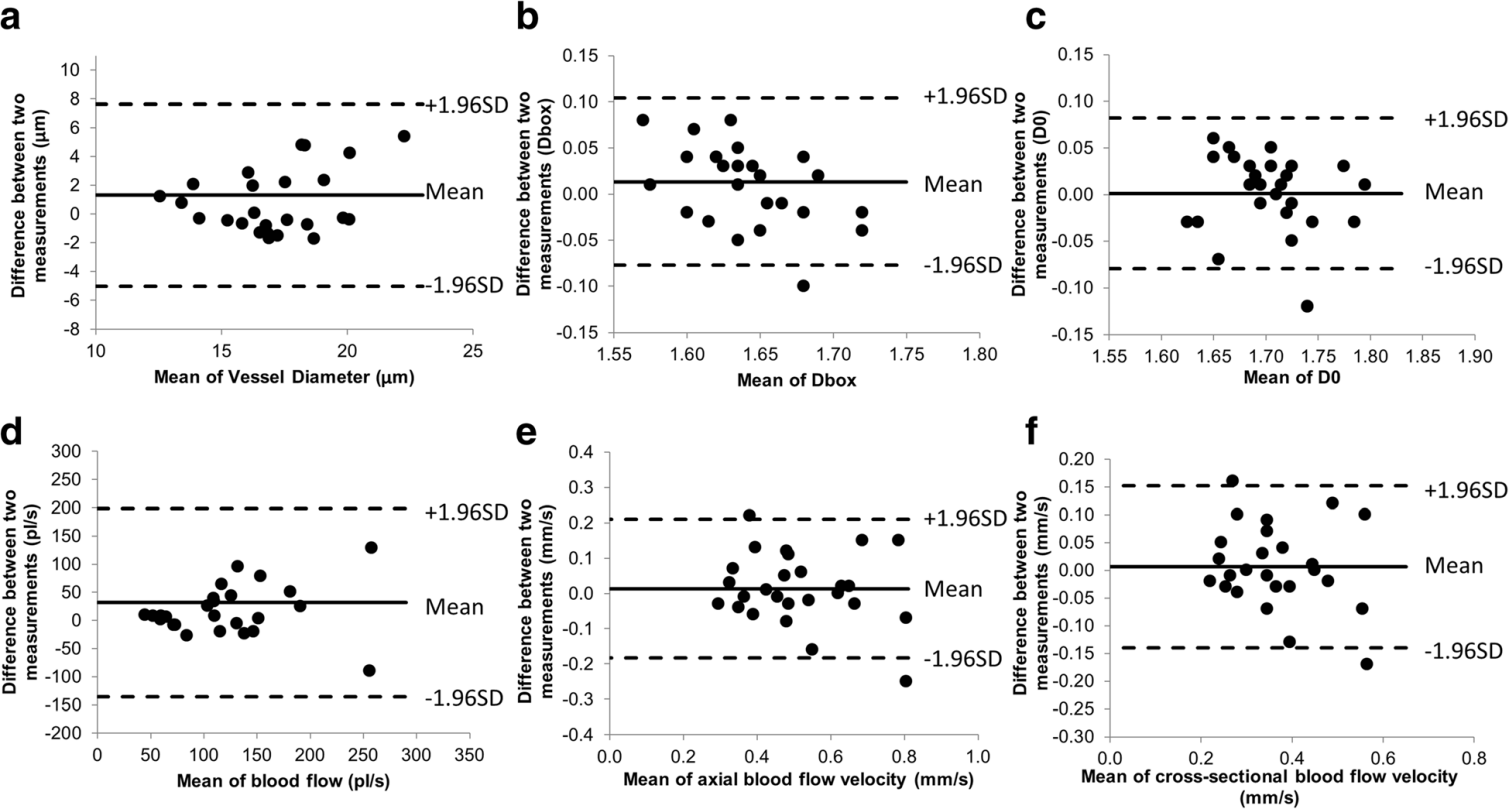
The Bland-Altman plots of habitual contact lens (HCL) wearers. The Bland-Altman plots in the HCL indicating the upper and lower limits of agreement of vascular measurements measured in both right and left eyes between two measurements taken in two visits, including vessel diameter (**a**), vessel density (Dbox, **b**), vessel complexity (D0, **c**), blood flow (**d**), axial blood flow velocity (**e**) and cross-sectional blood flow velocity (**f**)

**Fig. 4:**
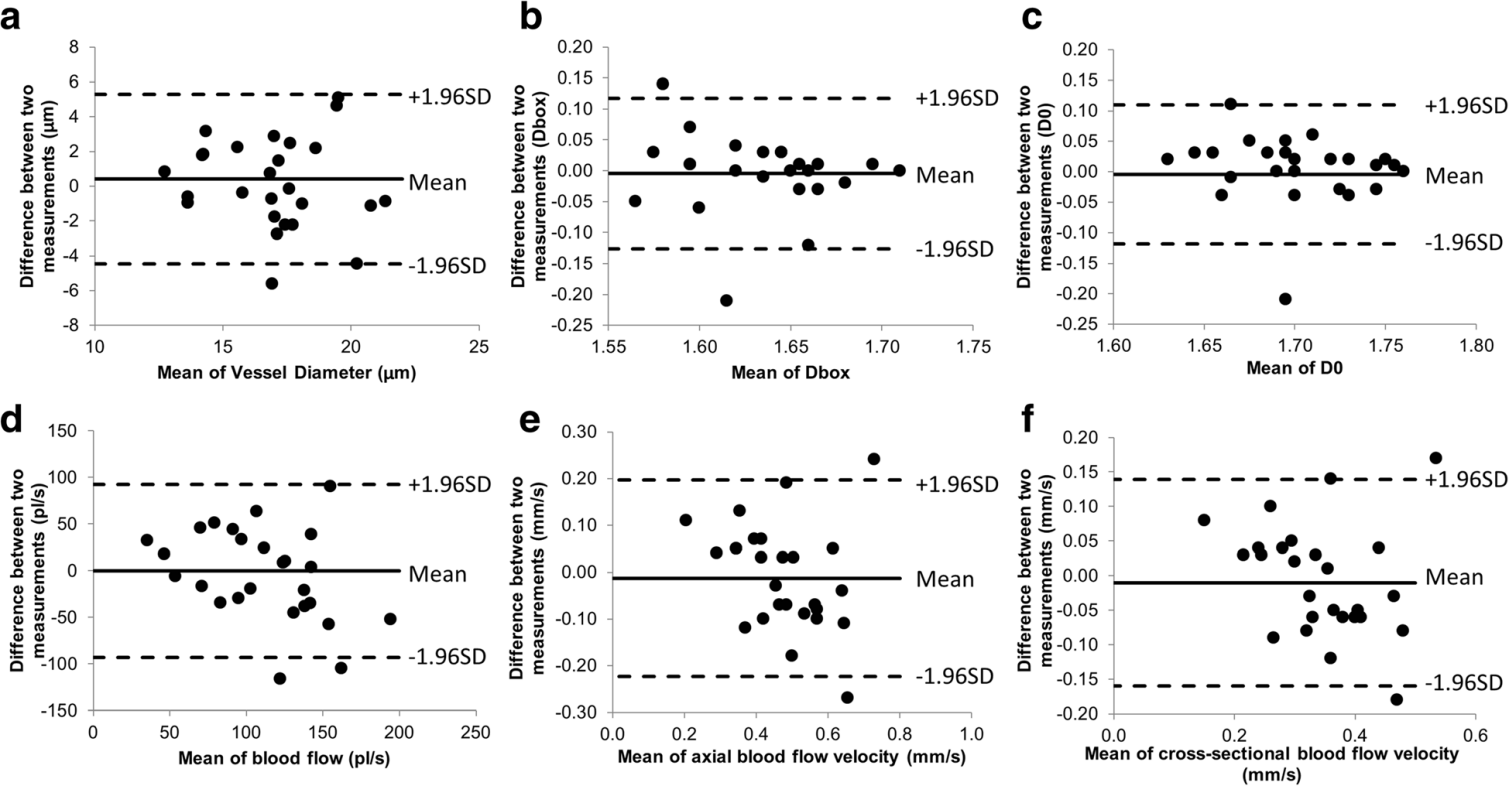
The Bland-Altman plots of non-contact lens (NCL) wearers. The Bland-Altman plots in the NCL indicating the upper and lower limits of agreement of vascular measurements measured in both right and left eyes between two measurements taken in two visits, including vessel diameter (**a**), vessel density (Dbox, **b**), vessel complexity (D0, **c**), blood flow (**d**), axial blood flow velocity (**e**) and cross-sectional blood flow velocity (**f**)

No significant differences of all vascular measurements in both the right and left eyes were found between two groups (*P* > 0.05, Table 1). The Bland-Altman plots in the HCL (Fig. 3) and NCL (Fig. 4) groups showed the upper and lower limits of agreement (i.e., ±1.96 SD, 95% confidence intervals). Between two measurements on two different visits, the CV ranged from 2.4% (vessel density D0) to 63.5% (blood flow volume, Q) in HCL wearers and from 3.4% (D0) to 40.6% (Q) in NCL wearers. The ICC was from 0.60 (vessel diameter) to 0.81 (VA) in HCL wearers and from 0.44 (Q) to 0.68 (cross-sectional blood flow velocity VS) in NCL wearers.

**Table 1:** Vascular measurements of HCL (*N* = 13) and NCL (*N* = 15) wearers in two visits

	D (μm)	Dbox	D0	Q (pl/s)	VA (mm/s)	VS (mm/s)
HCL						
Visit 1	18.1 ± 4.0	1.64 ± 0.04	1.70 ± 0.05	150 ± 119	0.52 ± 0.15	0.37 ± 0.10
Visit 2	16.8 ± 2.3	1.63 ± 0.06	1.70 ± 0.05	118 ± 62	0.51 ± 0.17	0.37 ± 0.12
Diff	1.3 ± 3.2	0.01 ± 0.05	0.00 ± 0.04	32 ± 85	0.01 ± 0.10	0.01 ± 0.07
Upper LOA	7.65	0.10	0.08	199	0.21	0.15
Lower LOA	−5.03	−0.08	− 0.08	− 135	− 0.18	−0.14
ICC	0.60	0.64	0.64	0.72	0.81	0.78
CV (%)	18.5	2.8	2.4	63.5	19.5	20.2
CI of CV	25.6~11.4	3.9~1.7	3.4~1.5	88.0~39.1	27.1~12.0	27.9~12.4
NCL						
Visit 1	17.2 ± 2.4	1.62 ± 0.06	1.69 ± 0.06	116 ± 43	0.49 ± 0.13	0.36 ± 0.10
Visit 2	16.8 ± 2.6	1.62 ± 0.06	1.70 ± 0.06	117 ± 53	0.51 ± 0.16	0.37 ± 0.12
Diff	0.4 ± 2.5	0.00 ± 0.06	0.00 ± 0.06	−0.2 ± 47	−0.01 ± 0.11	−0.01 ± 0.08
Upper LOA	5.3	0.12	0.11	93	0.20	0.14
Lower LOA	−4.5	−0.13	−0.12	−93	− 0.22	−0.16
ICC	0.51	0.49	0.51	0.44	0.67	0.68
CV (%)	14.7	3.8	3.4	40.6	21.4	21.1
CI of CV	20.3~9.0	5.3~2.4	4.8~2.1	56.3~25.0	29.6~13.2	29.2~13.0

*HCL* = habitual contact lens wearers; *NCL* = non-contact lens wearers; *D* = diameter; *Dbox* = fractal dimension processed using box counting indicating vessel density; D0 = multifractal dimension indicating vessel complexity; *Q* = blood flow volume; *VA* = axial blood flow velocity; *VS* = cross-sectional blood flow velocity; *CV* = coefficient of variation; *ICC* = interclass correlation coefficient; *LOA* = limit of agreement; *CI of CV* = confidence interval of CV

## Discussion

While the “true” values of these measured vascular parameters are unknown, the repeated measurements determine whether each of the measurements can be used interchangeably. In contact lens studies, repeated measurements are often needed when ocular responses to the lenses are being evaluated. Understanding the variation between repeated measurements will help interpret study results to determine whether the alteration is due to contact lens wear with different designs or materials. The CV of the blood flow velocity and vessel density found in this study is about the same as the changes due to contact lens wear documented in previous studies [6, 17], which provides a very good detection power (effect size = ~ 1) in determining whether the changes are true. This may explain the previous finding that significant changes were reached in a small cohort of contact lens wears compared to controls in neophytes [5] and habitual lens wearers [6]. However, whether these parameters have sufficient power in differentiating the vascular responses to different lens designs and materials remain unknown. Thus, further studies are warranted.

The Bland-Altman plots showed all dots within the 95% limits of agreement for vessel diameter and blood flow in the HCL, which may indicate good test-retest reliability in the HCL. Interestingly, the CV and ICC were about the same between HCL and NCL groups, indicating that both groups had similar variation between visits and the methodology used in the present study yielded the expected results. The inter-visit variation appears to be similar to intra-visit variation demonstrated by Xu et al., who tested these vascular parameters at 9 AM and 11 AM on the same day in a group of 20 healthy subjects, including habitual lens wearers and non-contact lens wearers [33]. In a recent inter-visit variability study conducted by Khansari et al., conjunctival hemodynamics of non-diabetic healthy subjects were measured repeatedly for 11 weeks, which was longer than the follow-up we did in the present study. Although a different image device was applied, the standard deviation of the difference between two visits was similar in conjunctival blood flow velocity (0.16 mm/s vs. 0.11 mm/s).

However, the CV of measuring blood flow velocity of the conjunctiva is higher than the CV of measuring retinal blood flow velocity [34]. Burgansky-Eliash et al. reported that the intra-visit variability of measuring retinal blood flow was 11.2%. The relatively large CV found in our study with two visits and the previous study with one visit for the measurements of blood flow velocity may be due to: 1) In fundamental ocular physiology, stable retinal blood flow is necessary to maintain the high metabolic activity of the retina for vision, whereas conjunctival blood flow is likely not as important for the conjunctiva. Further, retinal blood vessels are much more ordered than conjunctival blood vessels. Therefore, it is not surprising that conjunctival vessels have a larger CV; 2) the ocular surface exposure to the outside environment such as temperature which may affect the circulation of the conjunctiva; 3) blinking which may exert mechanical pressure on the vessels; and 4) the small volumes of blood flow velocity (about 0.50 mm/s) which is about 6–8 times slower than that in the retina (about 3–4 mm/s), and may lead to the higher CV results.

There are some limitations to the present study. First, the measurement of blood flow velocity is not fully automatic and required the manual drawing of the slope in the time-space images as performed in previous studies. The inter-grader variation of the blood flow velocity measurements is reported to be about 5% which may have been included in inter-visit variability. Therefore, further development of fully automated image processing for measuring the velocity is needed. Second, while attention is given to relocate the same image locations on the conjunctiva, actual fields of view were not obtained because it was nearly impossible to get the same location during a video recording using extremely high magnification. Although we tried to image vessels with similar diameters, the measurements were not controlled by the vessel diameters in the present study. Vessel diameter correlates to blood flow velocity [5, 35]. These factors in the selection of vessels and imaging location may contribute to the CV, and further development on field tracking may be helpful. Third, only the venules on the conjunctiva were measured. Since blood flow velocity is different between the arterioles and venules [6, 25, 26, 29, 30], further studies, including conjunctival arterioles, are needed. Last, the sample size may be small. Thirty subjects were recruited in total; however, two of them missed the second visit due to conflicts in their schedules. Nevertheless, this study provided reasonable results which corroborated with previous studies of intra-visit variability [33].

## Conclusion

In summary, the measurement variability of the vessel density of the bulbar conjunctiva appeared to have the smallest inter-visit variation. The measurement variability of the vasculature and circulation in HCL wearers were similar to that in NCL wearers. These findings are helpful in sample size calculation and study designs of further studies on conjunctival vascular responses to contact lens materials and designs.
